# Porous Talcum-Based Steatite Ceramics Fabricated by the Admixture of Organic Particles: Experimental Characterization and Effective Medium/Field Modeling of Thermo-Mechanical Properties

**DOI:** 10.3390/ma16124420

**Published:** 2023-06-15

**Authors:** Christian Pichler, Lukas Perfler, Roland Traxl, Roman Lackner

**Affiliations:** University of Innsbruck, Material Technology Innsbruck, Technikerstraße 13, A-6020 Innsbruck, Austria; lukas.perfler@uibk.ac.at (L.P.); roland.traxl@uibk.ac.at (R.T.); roman.lackner@uibk.ac.at (R.L.)

**Keywords:** steatite ceramics, Young’s modulus, bending strength, thermal conductivity, porosity, modeling

## Abstract

In this paper, an experimental campaign, as regards the thermo-mechanical properties (heat capacity, thermal conductivity, Young’s modulus, and tensile (bending) strength) of talcum-based steatite ceramics with artificially introduced porosity, is presented. The latter has been created by adding various amounts of an organic pore-forming agent, almond shell granulate, prior to compaction and sintering of the green bodies. The so-obtained porosity-dependent material parameters have been represented by homogenization schemes from effective medium/effective field theory. As regards the latter, thermal conductivity and elastic properties are well described by the self-consistent estimate, with effective material properties scaling in a linear manner with porosity, with the latter in the range of 1.5 vol-%, representing the intrinsic porosity of the ceramic material, to 30 vol-% in this study. On the other hand, strength properties are, due to the localization of the failure mechanism in the quasi-brittle material, characterized by a higher-order power-law dependency on porosity.

## 1. Introduction

Steatite is a constituent present in a variety of modern ceramic materials (see, e.g., [1,2]) and is frequently used as filler in composite materials (see, e.g., [3,4,5]). Steatite is characterized by low dielectric loss properties and high thermal and mechanical resistance. Combined with good workability and low shrinkage of the green bodies, steatite ceramics are predominantly used in electrical applications and as thermal insulation material. Recent, further applications were suggested in nuclear power applications [6] and medical engineering [7,8]. Recently, we described the synthesizing of a low-to-medium porosity talcum-based steatite (TBS) ceramic including a detailed pore space characterization and analysis of solid phase microstructure and composition [9]. Ceramic materials of this type are frequently denoted as steatite or steatite ceramic in the open literature. This may be ambiguous, as both the sintered product and the main raw material (talcum, soapstone), respectively, are denoted as “steatite”. Hence, the denomination “talcum-based steatite (TBS) ceramic” is chosen in this paper. [9] also contains a detailed description of the pore-forming agent—almond shell granulate—that has been intermixed within the green bodies prior to compaction and sintering. The so-obtained artificially introduced porosity may foster novel applications of the well-established ceramic material, as thermo-mechanical properties may be tailored to customer’s specifications.

The current paper focuses on the experimental characterization and discusses microstructure-based modeling of thermo-mechanical properties: heat capacity, thermal conductivity, Young’s modulus, and tensile (bending) strength of prism-shaped and plate-shaped specimens (obtained by cutting off from these prisms), respectively, of the porous TBS ceramic samples investigated in [9]. Thereby, Youngs’s modulus and bending (tensile) strength were determined from three-point bending experiments; in addition, elastic behavior was also assessed by ultrasonic compressive wave speed measurements in the longitudinal direction of the prisms. For the determination of thermal properties in the scopes of the laser flash method, the plate-shaped specimens were employed.

The material dealt with in this paper is characterized by a maximum porosity of about 30%. Hence, as regards modeling, we restrict the discussion to analytical modeling schemes pertinent for the low and medium porosity range. As regards modeling of thermo-mechanical properties of highly-porous materials, see, e.g., [10,11,12,13].

## 2. Material and Sample Preparation

For the manufacture of the dense ceramic samples, a commercially available granulated barium/talcum Mg${}_{3}$(Si${}_{4}$O${}_{10}$)(OH)${}_{2}$ mass (Quarzsandwerke Weißenbrunn, Bauer & Co., Weißenbrunn, Germany, labeled “C 221” and in accordance with IEC 672), composed by the raw materials, talcum, clay, and barium carbonate (with the latter serving as fluxing agent), was compacted (in dry state) with a force exceeding 50 kN (see Table 1) into green bodies (prisms with 10 mm × 10 mm × 120 mm) and sintered at temperatures between 1300 and 1350 °C for 2 h and subsequently cooled to 100 °C with a cooling rate of 100 K/h. The resulting TBS ceramic is composed of proto- and clinoenstatite crystals (Mg${}_{2}$Si${}_{2}$O${}_{6}$), i.e., pyroxenes, and a small amount of cristobalite, all embedded in a barium containing glassy phase. As described in detail in [9], XRD gave access to the crystalline mineral phases of the material (see Table 2, listing mass fractions related to the sum of crystalline phases only), with enstatite as the main phase. Additionally, a glassy phase, serving as a matrix in the material system, is present with a mass fraction of approximately 30 m% (related to total mass).

**Figure 1:**
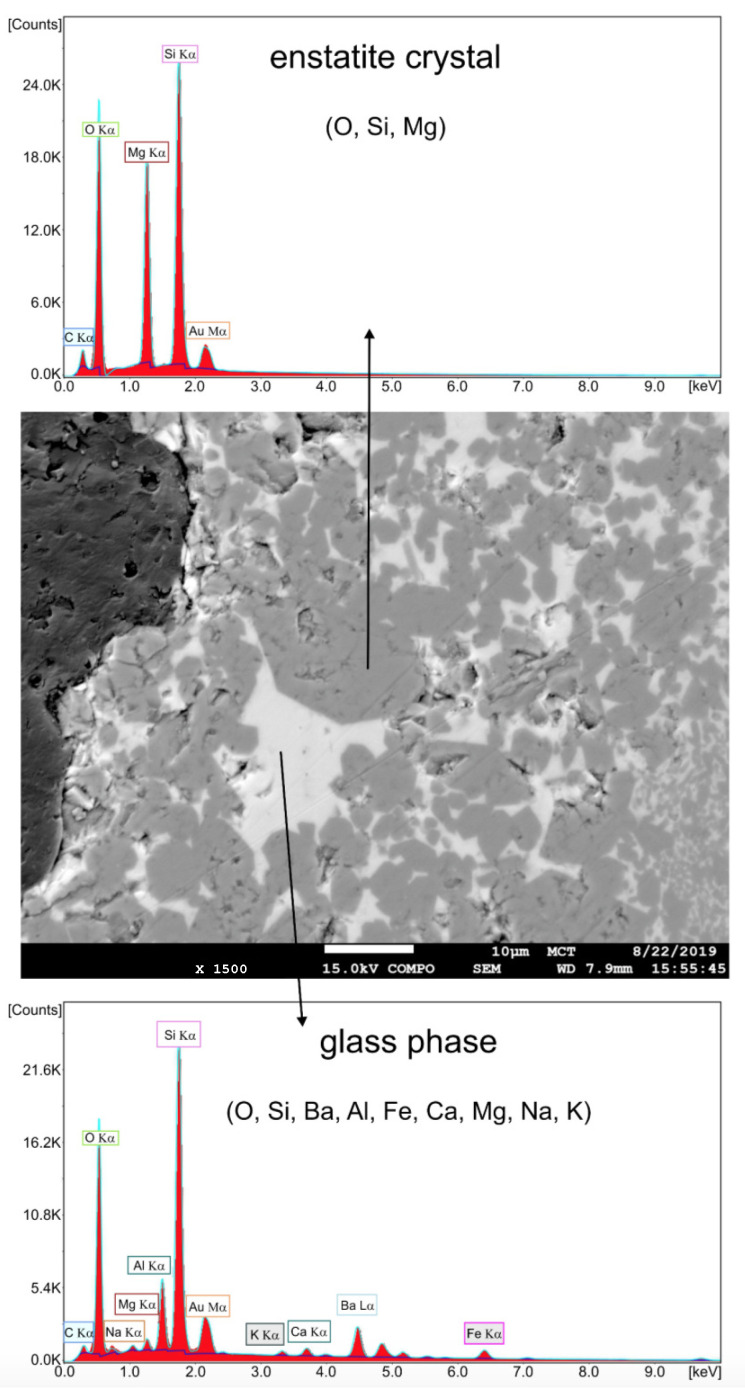
Backscattered electron microscopy image of a polished TBS ceramic sample (medium grey = enstatite crystals, light grey = glassy phase, dark grey = resin-filled pore) with associated EDX spectra; note: resin filling of pores was employed to facilitate polishing procedure.

High-resolution imaging combined with backscattered electron microscopy was employed to visualize the prevalent material phases, pore structure, and morphology. Chemical analyses of the TBS ceramic samples were obtained by energy dispersive X-ray (EDX) spectroscopy. Figure 1 shows the backscattered electron microscopy image of a polished sample together with associated EDX spectra. As indicated by the various grey scale values, TBS ceramic is composed by different material phases, mainly enstatite crystals (Mg${}_{2}$Si${}_{2}$O${}_{6}$, medium grey), embedded in a barium containing glassy phase (light grey); porosity is also visible (dark grey). A detailed chemical analysis of the glassy phase, containing mainly oxygen, silicon, barium, aluminum, and magnesium, can be found in [9].

**Figure 2:**
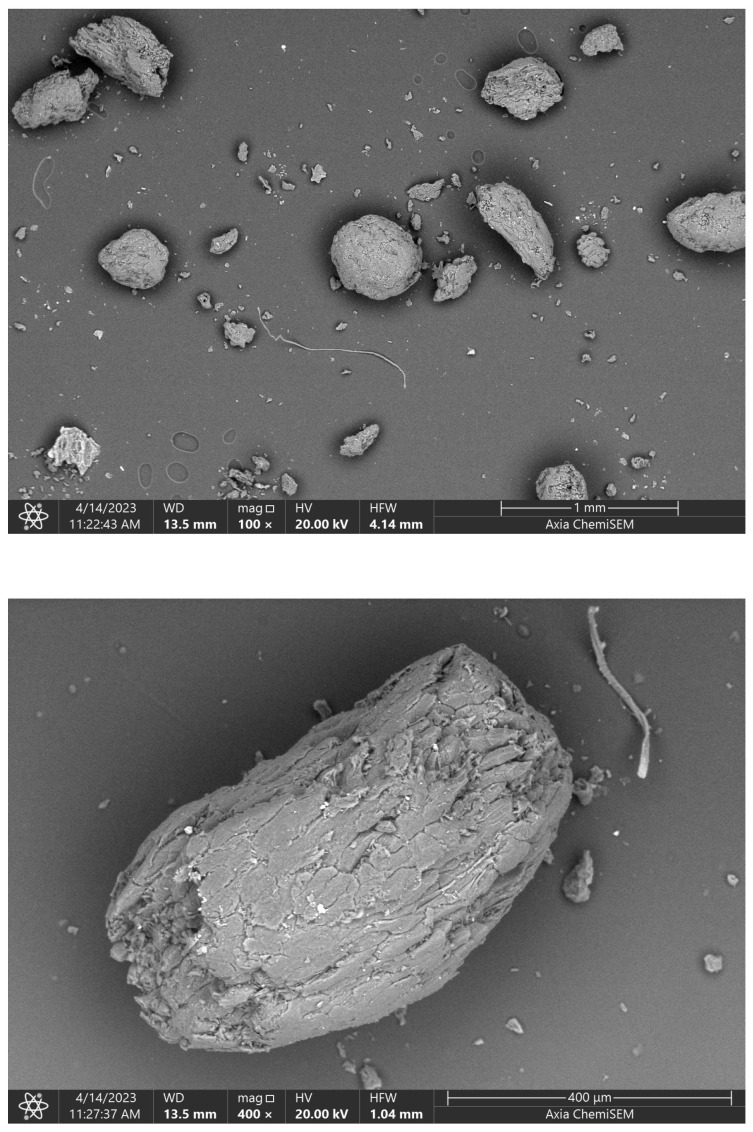
SEM images of organic material intermixed prior to compaction and sintering of green bodies for generation of artificial pore space: almond shell granulate (Rehofix MS 0–350, J. Rettenmaier and Söhne GmbH, Germany).

In addition to the dense samples (with their intrinsic porosity), same-size prismatic specimens with artificially introduced porosity were manufactured by intermixing organic particles in the green bodies prior to compaction and sintering (see Table 1). Almond shell granulate was employed as the pore-forming agent (Rehofix MS, diameter 0–200 μm and 0–350 μm, respectively, J. Rettenmaier and Söhne GmbH, Rosenberg, Germany) in various contents: 2, 4, 6, 8, and 10 m-%. SEM imaging (Figure 2) reveals the morphology of the pore-forming agent, and, hence, the morphology of the artificially introduced porosity in the sintered material. The particle shape ranges from few-micrometer-sized chips to hundred-micrometer, roughly equiaxed blocks. Looking forward to modeling, the granules may be approximated as oblate to prolate ellipsoids, respectively, with aspect ratios ranging from 1/4 to 2. Prior to thermo-mechanical characterization, the mass and geometric dimensions (length, width, and height) of the dried, prismatic ceramic specimens were determined with an analytical balance (Mettler Toledo ME204T) with an accuracy of 0.0001 g and a digital caliper (Preisser) with a resolution of ≥0.01 mm, respectively, giving access to the sample density $\rho_{\mathrm{eff}}$ (Table 3). The dense samples with intrinsic porosity (SC-0) exhibit an average density and mass of 2790 kg/m${}^{3}$ and 32.53 g, respectively. With an increase in artificially introduced porosity, the density and mass of the porous TBS ceramic prisms continuously decrease, with the porous samples SC-350-10 and SC-200-10 characterized by the lowest densities of 2110 and 2020 kg/m${}^{3}$, respectively.

In this paper, porosity of the (dried) sample $f_{p}$ [–], related to the volume fraction of the solid material matrix $f_{m}$ as $f_{p}=1-f_{m}$, is associated with a (medium) density of the solid material phases $\rho_{m}$ [kg/m${}^{3}$] (mainly protoenstatite, clinoenstatite, and the glassy phase, see Table 2), as $f_{m}=\left(1-f_{p}\right)=\rho_{eff}/\rho_{m}$, with $\rho_{eff}$ [kg/m${}^{3}$] denoting measured sample density. With an intrinsic porosity of the dense samples determined as $f_{p}=0.015$ in [9], $\rho_{m}$ = 2790/(1 − 0.015) = 2830 kg/m${}^{3}$. The latter value has been used in the figures throughout this paper to determine $f_{m}$ from measured sample densities.

## 3. Experimental Characterization

### 3.1. Thermal Properties

Laser flash analyses (LFA) gave access to the (effective) thermal diffusivity of the samples *D* [m${}^{2}$/s], with the latter reflecting both steady state heat flow, i.e., thermal conductivity *k* [W/(m K)], as well as thermal retention, i.e., (volume) heat capacity (ρc) [J/(m${}^{3}$K)], of a solid/porous material as D=k/(ρc). For an LFA measurement, one side of a disc or plate-shaped specimen with thickness *d* [m] is exposed to an energy pulse from a light source (laser or xenon flash lamp); on the opposite side of the specimen, the temperature history is measured [14,15,16]. The analytical solution of the related one-dimensional, adiabatic heat-transfer problem gives access to thermal diffusivity as $D=\delta d^{2}/t_{1/2}$ with δ=0.139 and $t_{1/2}$ denoting the time lag for the temperature to reach half of the final temperature rise. The higher the thermal diffusivity, the faster energy is dispersed within the sample.

**Figure 3:**
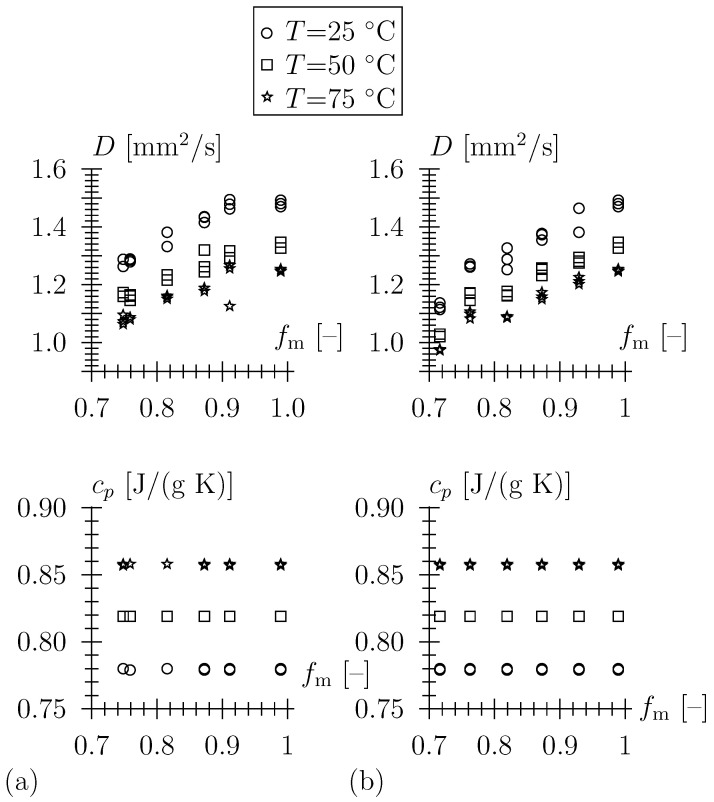
Experimental data obtained by LFA on porous ceramics, artificial porosity obtained by intermixing organic particles in green bodies prior to compaction and sintering, $f_{m}=\rho_{\mathrm{eff}}/\rho_{m}$ with $\rho_{m}$ = 2830 kg/m${}^{3}$, (**a**) pore-forming agent Rehofix MS 0–350, (**b**) pore-forming agent Rehofix MS 0–200.

A Netzsch LFA 447 NanoFlash system equipped with a Xenon flash lamp (λ = 150–2000 nm) and an infrared detector (InSb) with integrated dewar enabling measurements from room temperature up to 300 °C with a repeatability and accuracy of ±2–3% was employed in the present study. Plates with a thickness of 2 mm (surface area of 10 × 10 mm${}^{2}$) were cut from the ceramic prisms with a diamond saw. Thereafter, both surfaces of the cleaned and dried plates were coated with graphite spray twice (Graphit 33, Kontakt Chemie, CRC Industries Europe BV, Zele, Begium). For each graphite-coated sample, three laser flash shots were conducted at 25, 50, and 75 °C (see Figure 3a,b). At the lowest temperature, the dense TBS ceramic sample (SC-0 with $\rho_{\mathrm{eff}}$ = 2790 kg/m${}^{3}$) exhibits the highest thermal diffusivity with *D* = 1.48 mm${}^{2}$/s, and the sample with the highest porosity (SC-200-10, $\rho_{\mathrm{eff}}$ = 2020 kg/m${}^{3}$), with the lowest as 1.12 mm${}^{2}$/s. The specific heat capacity $c_{p}$ of the specimens was determined using a Pyroceram 9606 reference sample.

### 3.2. Mechanical Properties

Tensile bending strength was determined from the failure load in three-point experiments on the 120 mm long samples (span of 100 mm in bending experiments) with a cross-section of 10 × 10 mm${}^{2}$. Experiments were conducted on a Shimadzu AG-X 10 kN table-top model. This universal testing machine enables loading up to 10 kN. Data collection and assignment of the experimental conditions were accomplished by the TrapeziumX software (version 1.4.0). The specimens were loaded with a rate of 35 N/s until failure. These experiments also gave access to Young’s modulus as determined from the initial, linear part of the force-deflection graph.

Furthermore, elastic behavior was also assessed by ultrasonic (US) compressive wave speed measurements. Specimens with the same dimensions as in the bending experiments were employed, with US waves conveyed in the longitudinal direction of the specimens. For the propagation of elastic “bulk” compressive (or longitudinal) waves in an isotropic material (i.e., in the bulk continuum), the component $C_{\mathrm{iiii}}$ of the fourth order elasticity tensor is related to wave speed *v* [m/s] and density ρ [kg/m${}^{3}$] as [17]:

(1) $$C_{\mathrm{iiii}}=\frac{\left(1-\nu \right)}{\left(1+\nu )(1-2\nu \right)}E=\rho v^{2}$$

with *E* [Pa] and ν [–] denoting Young’s modulus and Poisson’s ratio, respectively. Thereby, the displacement of material points (particle polarization) and wave propagation direction is equi-directional.

This theory of bulk wave propagation still holds for pulse propagation through finite bars (in the longitudinal direction) in case the wavelength λ is much smaller than the cross-sectional dimension of the bar [18]. For the present investigation, the measured velocities ranged from 5050 to 6900 m/s and the frequency of the employed transducers is given as *f* = 220 kHz. Hence, the minimum wavelength is given as λ = 5050/220,000 = 30 × 10${}^{-3}$ m, i.e., the condition is not met with a cross-sectional dimension of 10 × 10${}^{-3}$ m. On the other hand, when λ≫ than the cross sectional dimension, “extensional” or “bar” wave propagation with a (as opposed to bulk wave propagation) one-dimensional state of normal stress is predominant with [19]:

(2) $$v=\sqrt{\frac{E}{\rho }}$$

In the present case, λ = 30 mm is, to an extent, larger than the cross-sectional dimension of 10 mm, and Equation (2) was employed for the backcalculation of Young’s modulus from ultrasonic compressive wave speed measurements.

## 4. Discussion: Effective Medium/Field Modeling of Structure/Property-Relations

### 4.1. Thermal Conductivity

**Figure 4:**
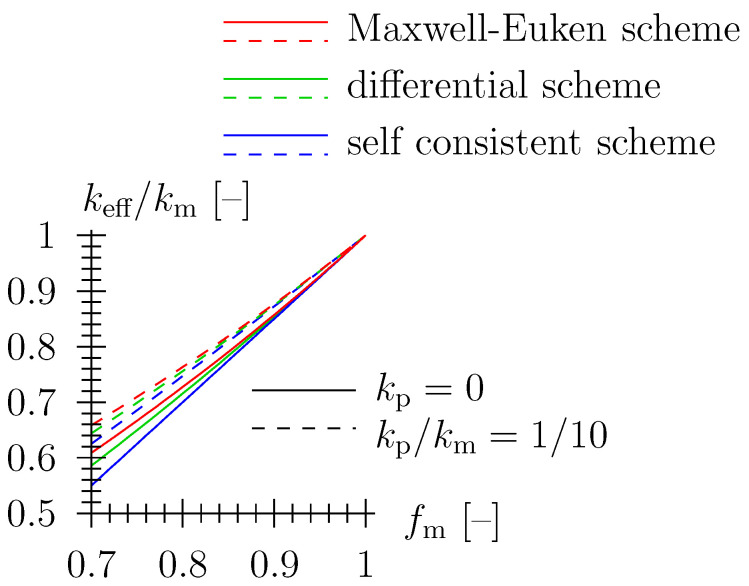
Prediction of classical homogenization schemes for spherical pore shape; as regards porous ceramics, the assumption of $k_{p}\approx 0$ is usually acceptable as the contrast between conductivity of matrix material and pore space, $k_{m}$ and $k_{p}$, respectively, is large, i.e., $k_{p}/k_{m}<1/100$.

Well established schemes from effective media and effective field theory [20] may be employed to relate the thermal conductivity of the porous material $k_{\mathrm{eff}}$ [W/(m K)] to the conductivity and volume fraction of the solid material matrix, $k_{m}$ [W/(m K)] and $f_{m}$ [–], respectively (for details, see Appendix A). Pore space morphology may be taken into account, with extensions of classical schemes accessible for spheroidal pore shapes (for details, see Appendix B). Hence, $k_{\mathrm{eff}}=k_{\mathrm{eff}}\left(k_{m},f_{m}=1-f_{p},\mathrm{morphology}\right)$. When accounting for (i) matrix/spherical pore morphology and (ii) zero conductivity in the pore space, where the latter is a reasonable simplification in case the contrast between conductivity of matrix material and pore space, $k_{m}$ and $k_{p}$, respectively, is large (that is the case for most ceramic materials), engineering approximations are available, as listed below (see also, Figure 4). As regards porous ceramics, the assumption of $k_{p}\approx 0$ is usually acceptable, as the contrast between conductivity of matrix material and pore space, $k_{m}$ and $k_{p}$, respectively, is large, i.e., $k_{p}/k_{m}<1/100$. For porous TBS ceramics, with $k_{p}$ = 0.018 W/(m K) as the respective value for air, the contrast is approximately 0.018/3 = 1/170.

**Figure 5:**
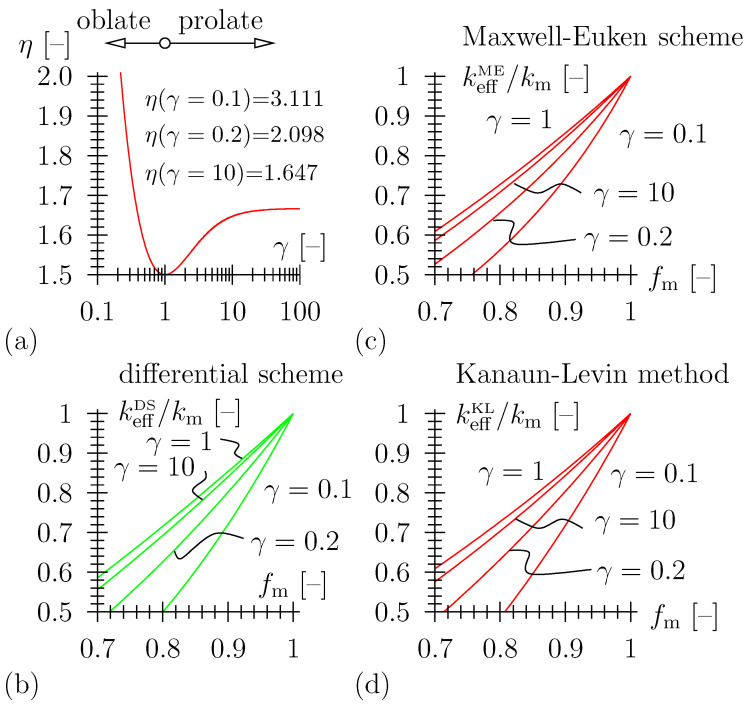
(**a**) Shape factor η as a function of aspect ratio γ according to [20,36], (**b**) consideration of pore shape for differential scheme, $k_{p}=0$, (**c**) consideration of pore shape for Maxwell–Eucken scheme, and (**d**) Kanaun–Levin method, $k_{p}=0$; for γ=1, Maxwell–Eucken scheme and Kanaun–Levin method coincide.

- Maxwell–Eucken expression [21], Kanaun–Levin method [22,23,24,25], or Hashin–Shtrikman upper bound [26] (in continuum micromechanics, the related homogenization method is referred to as the Mori–Tanaka scheme [27,28], see Appendix A):
  (3) $$\frac{k_{\mathrm{eff}}^{\mathrm{ME}}}{k_{m}}=\frac{k_{\mathrm{eff}}^{\mathrm{KL}}}{k_{m}}=\frac{2f_{m}}{3-f_{m}},$$
  derived for a spatial configuration where the local temperature distortion in the vicinity of a pore does not affect the temperature field in the vicinity of neighboring pores [29,30]. The expression may be employed for moderate porosity values, e.g., for spherical pores for $f_{p}<∼$0.2.
- The differential scheme (DS) [31,32,33] departs from this restriction, i.e., is suitable for materials with porosity in the medium range. This scheme follows from a recurring application of the dilute distribution estimation [34,35], the latter representing a spatial configuration where pores are diluted in the matrix material and, hence, their interaction can be neglected; starting with the homogeneous matrix material, pore volume is embedded in the matrix material in infinitesimal steps and the dilute distribution estimation is employed for the determination of effective behavior in each step, etc., finally giving:
  (4) $$\frac{k_{\mathrm{eff}}^{\mathrm{DS}}}{k_{m}}=f_{m}^{3/2}.$$
- One may consider spheroidal pore shape for the Maxwell–Eucken expression, the Kanaun–Levin method, and the differential scheme, with the influence of oblate and prolate geometry on effective behavior derived in [20,36]. For an isotropic distribution of spheroidal pores, the differential scheme reads:
  (5) $$\frac{k_{\mathrm{eff}}^{\mathrm{DS}}}{k_{m}}=f_{m}^{\eta },$$
  (see Figure 5b) with shape factor η≥3/2 a function of the aspect ratio γ of the spheroidal pores and γ<1 corresponding to oblate, γ>1 to prolate pore shape, respectively (see Appendix B). Figure 5a depicts the shape factor η as a function of aspect ratio γ. Note that whereas a prolate shape only minorly influences the shape factor, oblate shape with γ<0.2 significantly alters the effective thermal behavior (as compared to a composite with spherical pores), as η gets excessively big. Even a small porosity may lead to a significant reduction of thermal conductivity.
  Further note that for non-spherical pore shape, the Kanaun–Levin method and the Maxwell–Euken scheme do not coincide (for spherical pores, they do), with:
  (6) $$\frac{k_{\mathrm{eff}}^{\mathrm{KL}}}{k_{m}}=\frac{1-2/3\eta (1-f_{m})}{1+1/3\eta (1-f_{m})}$$
  and
  (7) $$\frac{k_{\mathrm{eff}}^{\mathrm{ME}}}{k_{m}}=\frac{f_{m}}{f_{m}\left(1-\eta \right)+\eta },$$
  respectively (see Figure 5c,d).
- The self-consistent scheme (SCS) [37,38] has originally been derived for so-called polycrytalline microstructures, where none of the material phases play a distinguished role. If one material phase is represented by porosity with $k_{p}=0$, the SCS reads:
  (8) $$\frac{k_{\mathrm{eff}}^{\mathrm{SCS}}}{k_{m}}=\frac{3f_{m}-1}{2}.$$

**Figure 6:**
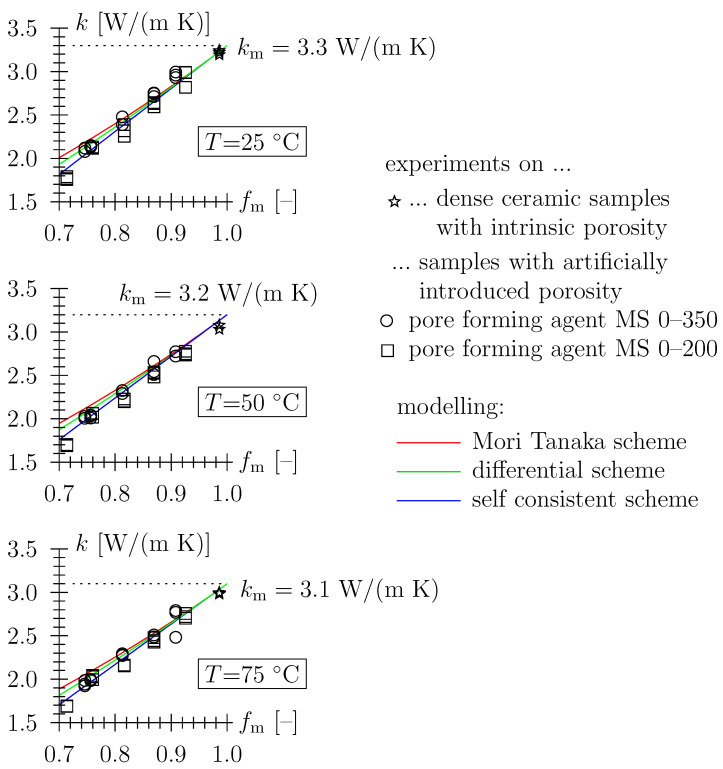
Thermal conductivity of porous ceramics, artificial pores obtained by intermixing organic particles (almond shell granulate) in the green bodies prior to compaction and sintering; comparison between experimental data with $f_{m}=\rho_{\mathrm{eff}}/\rho_{m}$ and $\rho_{m}$ = 2830 kg/m${}^{3}$ and micromechanical modeling ($k_{p}=0$, spherical pore shape).

Based on the measured diffusivity and heat capacity (see Section 3.1), the thermal conductivity is obtained as k=D/(ρc) and plotted as a function of solid matrix volume fraction (see Figure 6). When assessing the representation of data for the three major homogenization schemes described previously (ME, DS, SCS; spherical pore shape), one may select the SCS as the most appropriate scheme. However, the scatter of data, with the variation obtained in this paper in the typical range for ceramic materials, makes that choice rather sketchy. Also note that for a given porosity, i.e., $f_{m}=$ const., thermal conductivity decreases with temperature (in the investigated temperature range from 25 to 75 °C). The (theoretical) value for the solid phase conductivity may be approximated to decrease from 3.3 to 3.1 W/(m K) in this temperature range (see Figure 6). Data quality also prevents one from backcalculating an (average) pore shape factor based on Equations (5)–(7). The average shape of the artificial pores seems to be too compact, or equiaxed, to have a significant influence on the shape of the material function $k_{\mathrm{eff}}/k_{m}=k_{\mathrm{eff}}/k_{m}\left(f_{m}\right)$.

### 4.2. Mechanical Properties

**Figure 7:**
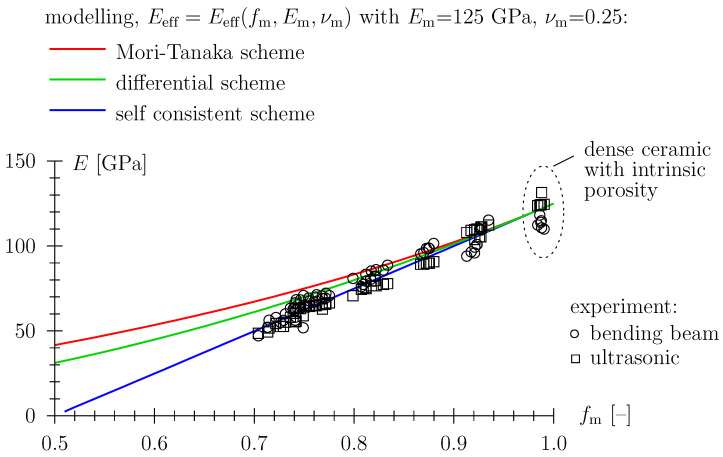
Young’s modulus of porous TBS ceramics; $f_{m}=\rho_{\mathrm{eff}}/\rho_{m}$ with $\rho_{m}$ = 2830 kg/m${}^{3}$, comparison with models from effective medium theory.

Figure 7 depicts a comparison of experimental data for Young’s modulus (see Section 3.2) with classical schemes from effective medium theory (see Appendix A), with the self-consistent scheme giving a fairly reasonable representation of data.

As regards the modeling of input parameters, spherical pore shape is underlain, as well as the density and elastic properties of the solid material phase, summarized in Figure 7.

In order to employ the self-consistent scheme for upscaling of strength, further assumptions are necessary. (i) For incompressible matrix behavior, i.e., $\mu_{m}=E_{m}/3$, with $\mu_{m}$ denoting shear modulus of the matrix material, the self-consistent estimate can be written as:

(9) $$\begin{array}{ccccc} \mu_{\mathrm{eff}} & = & M\left(f_{m}\right)\mu_{m} & = & \frac{3(2f_{m}-1)}{2+f_{m}}\mu_{m}\;\;\;\mathrm{and}\\ K_{\mathrm{eff}} & = & K\left(f_{m}\right)\mu_{m} & = & \frac{4\left(2f_{m}-1\right)f_{m}}{\left(1-f_{m}\right)\left(2+f_{m}\right)}\mu_{m}\;. \end{array}$$

(ii) For ductile, von Mises-type behavior of the matrix material, with $c_{m}$ as the cohesion of the matrix material, the domain of admissible macroscopic stress states is given as (see Equation (7.70) in [39]):

(10) $$f_{m}=\frac{1}{K(f_{m})}{\left(\frac{\Sigma_{\mathrm{vol}}/3}{c_{m}}\right)}^{2}+\frac{1}{M(f_{m})}{\left(\frac{\Sigma_{\mathrm{dev}}}{c_{m}}\right)}^{2}$$

where $\Sigma_{\mathrm{vol}}$ and $\Sigma_{\mathrm{dev}}$ denote the volumetric and deviatoric macroscopic stress, respectively. Setting $\Sigma_{\mathrm{vol}}$=0, the effective cohesive strength $c_{\mathrm{eff}}=\Sigma_{\mathrm{dev}}\left(\Sigma_{\mathrm{vol}}=0\right)$ is obtained as:

(11) $$\frac{c_{\mathrm{eff}}}{c_{m}}=\sqrt{\frac{3f_{m}\left(2f_{m}-1\right)}{2+f_{m}}}$$

**Figure 8:**
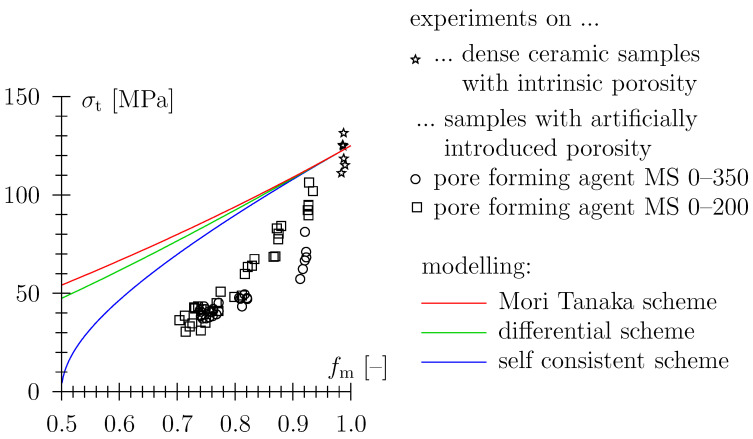
Bending strength of porous TBS ceramics; $f_{m}=\rho_{\mathrm{eff}}/\rho_{m}$ with $\rho_{m}$ = 2830 kg/m${}^{3}$, comparison with self consistent estimate and other models from effective medium theory.

Assuming that the (tensile) bending strength $\sigma_{t}$ scales in the same way as cohesion, Figure 8 shows the self-consistent estimate by the scaling matrix strength of 125 MPa with the r.h.s of Equation (11). The model crudely overestimates data (see Figure 8). Employing the reasoning from Equations (9)–(11) for the Mori–Tanaka schemes leads with $M\left(f_{m}\right)=\mu_{\mathrm{eff}}/\mu_{m}=f_{m}/\left(1+2/3\left(1-f_{m}\right)\right)$ to $c_{\mathrm{eff}}/c_{m}=f_{m}/\sqrt{1+2/3(1-f_{m})}$, see Figure 8, see also [40]. For the differential scheme, M has to be determined numerically and $c_{\mathrm{eff}}/c_{m}$ is determined as $\sqrt{f_{m}M}$. For the green graph in Figure 8, $M(f_{m})$ has been determined for a Poisson’s ratio of ν = 0.4999.

**Figure 9:**
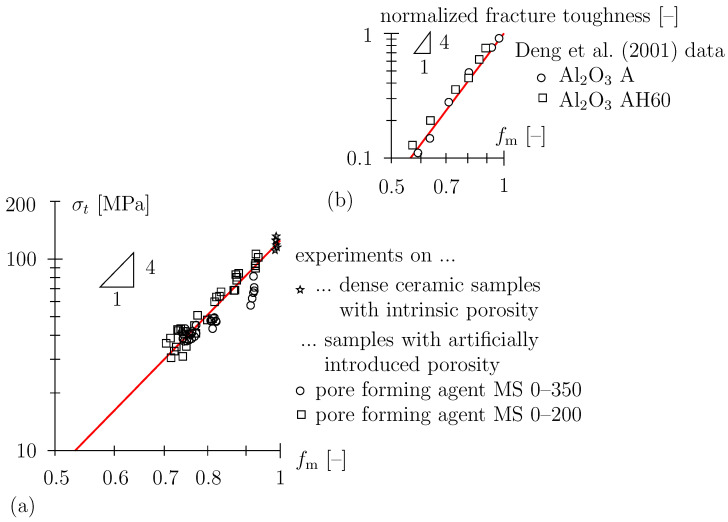
(**a**) Bending strength of porous TBS ceramics; $f_{m}=\rho_{\mathrm{eff}}/\rho_{m}$ with $\rho_{m}$ = 2830 kg/m${}^{3}$, red graph shows power-law approximation $\sigma_{t,\mathrm{eff}}=\sigma_{t,m}f_{m}^{4}$ with $\sigma_{t,m}$ = 125 MPa, (**b**) normalized fracture toughness (i.e., relative to the dense material) of porous ceramics, data taken from [41] (as cited in [42,43]).

This discrepancy may be explained by the localized brittle failure behavior of the material. When plotting data in a double-logarithmic diagram (Figure 9a), a power law approximation with bending strength proportional to the fourth power, the solid volume fraction of the solid material matrix, $\sigma_{t,\mathrm{eff}}\propto f_{m}^{4}$, reasonably represents data. Thereby, the strength of the solid phase $\left(f_{m}=1\right)$ is given as approximately $\sigma_{t,m}$ = 125 MPa. A power-law dependency with an exponent of approximately 4 has also been reported previously for data on the fracture toughness of porous alumina ceramics [41], see Figure 9b.

The influence of spheroidal pores on fracture behavior of brittle materials may be characterized by the stress concentration factor *p* relating a far field uniaxial (tensile) stress to the stress in circumferential direction (so-called hoop stress) in the vicinity of a spherical (or ellipsoidal) pore. For a spherical pore, the maximum tensile stress is approximately twice the far field stress, i.e., p≈2 [44], see Appendix C. The stress concentration factor has been identified as the major parameter influencing fracture strength of brittle materials with a power-law scaling function [45]:

(12) $$\frac{\sigma_{t,\mathrm{eff}}}{\sigma_{t,m}}={\left(1-f_{p}\right)}^{p}={\left(f_{m}\right)}^{p}.$$

As regards ellipsoidal pores, ref. [45] investigated oblate and prolate shapes. Extreme stresses are associated with the maximum mean curvature of the pore surface which are located at the poles (prolate) or the equator (prolate) of the ellipsoidal pores. The stress concentration factor *p* may be, also depending on the inclination of the axes of the ellipsoid with regards to the direction of the far field stress, larger (and also smaller) than two. For example, for an oblate pore with an aspect ratio of 0.1 with poles aligned in the direction of the far field stress, *p* is approximately 13.5. Stress concentration factors have also been reported for the case that the rotational axis of the spheroidal pore (axis running through poles) is inclined with an angle 0≤α≤π with regard to the direction of the far field stress [45]. In case of an assembly of ellipsoidal pores with uniform geometry randomly positioned in the solid material (with regard to inclination of ellipsoid axes), pores, however, separated sufficiently for limited mutual interaction (as regards the locally disturbed stress/strain fields); an effective angle of α = 54° represents the assembly [45,46]. For oblate pore shape and the mentioned effective angle, a stress concentration factor *p* of approximately four, which is what has been observed in our experimental program (see Figure 9a), is obtained for an aspect ratio between 0.1 and 0.2 (see Figure 1a in [45]).

## 5. Concluding Remarks

In this paper, the thermo-mechanical properties of talcum-based steatite (TBS) ceramics with artificially introduced porosity have been (i) characterized experimentally and (ii) represented by homogenization schemes from effective medium/effective field theory. Porosity ranged from 1.5 vol-%, the intrinsic porosity of TBS ceramics, to 30 vol-% in this study. Thermal conductivity and elastic properties are well described by the self-consistent estimate, with effective material properties scaling in a linear manner with porosity as $\propto (1-f_{p})$. By contrast, the strength properties are, due to the localization of the failure mechanism in the quasi-brittle material, characterized by a higher-order power-law dependency with strength scaling with a proportionality of $\propto {\left(1-f_{p}\right)}^{4}$. The latter dependency has been reasoned by the stress concentration factor for spheroidal pore shapes.

Future work will be devoted to the microstructural characterization of the solid material phases (protoenstatite, clinoenstatite, cristobalite, and the glassy phase) as regards their morphology and micro-mechanical behavior, e.g., via nanoindentation. This may allow the application of more advanced multiscale models for the prediction of thermo-mechanical properties of the sintered material [47,48].

## Figures and Tables

**Table 1:** Sample notation and preparation.

Sample Notation	Organic Material Added Prior to Compaction and Sintering	Organic Content [m-%]	Sample Compaction Force [kN]
SC-0	none, dense sample	0	50
	with intrinsic		
	porosity		
SC-350-02	Rehofix MS 0–350 μm	2	65
SC-350-04	Rehofix MS 0–350 μm	4	69
SC-350-06	Rehofix MS 0–350 μm	6	71
SC-350-08	Rehofix MS 0–350 μm	8	88
SC-350-10	Rehofix MS 0–350 μm	10	95
SC-200-02	Rehofix MS 0–200 μm	2	73
SC-200-04	Rehofix MS 0–200 μm	4	82
SC-200-06	Rehofix MS 0–200 μm	6	88
SC-200-08	Rehofix MS 0–200 μm	8	95
SC-200-10	Rehofix MS 0–200 μm	10	106

**Table 2:** Ceramic composition (m%) as determined by XRD (crystalline phases only).

protoenstatite	75.38
clinoenstatite	22.85
cristobalite	1.77
	100.00

**Table 3:** Mean values of length, width, height, mass, and sample density of investigated ceramic samples.

Sample	Length	Width	Height	Mass	$\rho_{\mathrm{eff}}$
	(mm)	(mm)	(mm)	(mm)	(kg/m${}^{3}$)
SC-0	119.55	9.78	9.99	32.53	2790
SC-350-02	119.54	10.00	10.04	30.86	2570
SC-350-04	119.70	10.02	10.14	29.88	2460
SC-350-06	119.60	10.01	10.13	27.85	2300
SC-350-08	120.26	10.07	9.99	26.05	2150
SC-350-10	120.43	10.09	10.03	25.65	2110
SC-200-02	120.04	10.03	9.82	30.94	2620
SC-200-04	120.17	10.04	9.93	29.51	2460
SC-200-06	120.17	10.05	9.97	27.79	2310
SC-200-08	120.62	10.08	10.12	26.48	2150
SC-200-10	120.91	10.10	10.23	25.18	2020

## Data Availability

Data will be made available on request.

## Appendix A. Homogenization Schemes for Elastic and Transport Properties of Two-Phase Composite Material (Effective Medium Theory)

Homogenization schemes from continuum micromechanics provide a sound modeling framework and determine effective the elastic properties or effective thermal conductivity of two-phase composite materials. In the former case, material tensors $M_{i}$, $M_{m}$, and $M_{\mathrm{eff}}$ in this appendix are replaced by the elasticity tensors of inclusion phase $C_{i}$, matrix material $C_{m}$, and homogenized or effective material $C_{\mathrm{eff}}$. For upscaling of the thermal conductivity, assuming isotropic behavior, $M_{i}$, $M_{m}$, and $M_{\mathrm{eff}}$ are replaced by the (scalar values) of the thermal conductivity of inclusion material phase $k_{i}$, matrix material $k_{m}$, and homogenized or effective material $k_{\mathrm{eff}}$. Moreover, the Eshelby tensor $S_{m}$ employed within the framework of continuum micromechanics reduces to the scalar value of 1/3 for spherical inclusions.

The dilute distribution (DD) estimation follows from the the Eshelby solution [34] as:

(A1) $$M_{\mathrm{eff}}=M_{m}+f_{i}\left(M_{i}-M_{m}\right):A_{i}^{\infty }\;\mathrm{with}\;A_{i}^{\infty }={\left[I+S_{m}:M_{m}^{-1}:\left(M_{i}-M_{m}\right)\right]}^{-1}\;,$$

where $f_{i}$ denotes the volume fraction of the inclusion phase, giving the volume fraction of the matrix phase as $f_{m}=1-f_{i}$.

The differential scheme (DS) represents a recursive formulation of the dilute distribution estimation. Starting with the homogeneous matrix material $M_{m}$, the inclusion phase with volume fraction $f_{i}$ is embedded in the matrix material in infinitesimal steps $df_{i}$. After each of these steps, the behavior of the matrix phase is updated based on the dilute distribution estimation ((n+1)-st step shown):

(A2) $$\begin{array}{ccc} \frac{dM_{\mathrm{eff}}^{n+1}\left(f_{i}^{n+1}\right)}{df_{i}} & = & \frac{M_{\mathrm{eff}}^{n+1}-M_{\mathrm{eff}}^{n}}{df_{i}}\\ \; & = & \frac{1}{1-f_{i}^{n}}\left(M_{i}-M_{\mathrm{eff}}^{n}\left(f_{i}^{n}\right)\right):\\ \; & \; & {\left[I+S_{\mathrm{eff}}^{n}\left(f_{i}^{n}\right):{\left(M_{\mathrm{eff}}^{n}\left(f_{i}^{n}\right)\right)}^{-1}:\left(M_{i}-M_{\mathrm{eff}}^{n}\left(f_{i}^{n}\right)\right)\right]}^{-1}, \end{array}$$

giving $M_{\mathrm{eff}}^{n+1}=M_{\mathrm{eff}}^{n}+dM_{\mathrm{eff}}^{n+1}$, using the initial condition $M_{\mathrm{eff}}\left(f_{i}=0\right)=M_{m}$.

The Mori–Tanaka (MT) scheme assumes that in a sufficient distance from an inclusion, the strain field (or temperature gradient) can be approximated by the volumetric average of the strain field over the entire matrix domain; hence, it is valid for a limited value of the inclusion volume fraction (for spherical inclusions approximately 0.2 or smaller):

(A3) $$M_{\mathrm{eff}}=M_{m}+f_{i}\left(M_{i}-M_{m}\right):{\left[I+\left(1-f_{i}\right)S_{m}:M_{m}^{-1}:\left(M_{i}-M_{m}\right)\right]}^{-1}\;.$$

In the scope of the self-consistent scheme (SCS), suitable for a polycrystalline microstructure with none of the material phases playing a specific morphological role, both material phases are modeled to be embedded in the sought-for homogenized material, leading to the implicit equation:

(A4) $$M_{\mathrm{eff}}=M_{m}+f_{i}\left(M_{i}-M_{m}\right):{\left[I+S_{\mathrm{eff}}:M_{\mathrm{eff}}^{-1}:\left(M_{i}-M_{\mathrm{eff}}\right)\right]}^{-1}\;.$$

Recently, the listed homogenization schemes have been unified in the scope of the cascade micromechanical model [49,50,51,52], accomplishing a continuous transition between schemes via the cascade order: for a cascade order of one, the Mori–Tanaka scheme is recovered; for an order of two, the differential scheme; and finally, the cascade order →∞ yields the prediction of the self-consistent scheme.

### Specialization for Effective Thermal Conductivity of Porous Materials

With volume fractions of solid matrix material and pores, $f_{m}$ and $f_{p}=f_{i}$, respectively, $f_{m}+f_{p}=1$ and the related thermal conductivities $k_{m}$ and $k_{p}$, and, further, considering the material isotropy and spherical shape of pores, the Mori–Tanaka estimate for the effective conductivity is given as:

(A5) $$k_{\mathrm{eff}}^{\mathrm{MT}}=k_{m}+\frac{f_{p}\left(k_{p}-k_{m}\right)}{1+\left(1-f_{p}\right)\frac{1}{3}\frac{k_{p}-k_{m}}{k_{m}}}$$

For the differential scheme, a closed form solution cannot be given when $k_{p}>0$; the solution needs to be obtained numerically from:

(A6) $$\frac{d{\left(k_{\mathrm{eff}}^{\mathrm{DS}}\right)}^{n+1}}{d{\left(f_{p}\right)}^{n+1}}=\frac{1}{1-{\left(f_{p}\right)}^{n}}\frac{k_{p}-{\left(k_{\mathrm{eff}}^{\mathrm{DS}}\right)}^{n}}{1+\frac{1}{3}\frac{k_{p}-{\left(k_{\mathrm{eff}}^{\mathrm{DS}}\right)}^{n}}{{\left(k_{\mathrm{eff}}^{\mathrm{DS}}\right)}^{n}}}$$

and

(A7) $${\left(k_{\mathrm{eff}}^{\mathrm{DS}}\right)}^{n+1}={\left(k_{\mathrm{eff}}^{\mathrm{DS}}\right)}^{n}+d{\left(k_{\mathrm{eff}}^{\mathrm{DS}}\right)}^{n+1}$$

with initial condition ${\left(k_{\mathrm{eff}}^{\mathrm{DS}}\right)}^{0}=k_{m}$.

For the self consistent scheme, the implicit equation:

(A8) $$k_{\mathrm{eff}}^{\mathrm{SCS}}=k_{m}+\frac{f_{p}\left(k_{p}-k_{m}\right)}{1+\frac{1}{3}\frac{k_{p}-k_{\mathrm{eff}}^{\mathrm{SCS}}}{k_{\mathrm{eff}}^{\mathrm{SCS}}}}$$

is solved as:

(A9) $$k_{\mathrm{eff}}^{\mathrm{SCS}}=\frac{1}{4}\left[k_{m}\left(2-3f_{p}\right)+k_{p}\left(3f_{p}-1\right)\pm \sqrt{8k_{m}k_{p}+{\left[k_{m}\left(3f_{p}-2\right)+k_{p}\left(1-3f_{p}\right)\right]}^{2}}\right]\;.$$

The specialization of these three schemes for zero conductivity of porous space is summarized as:

(A10) $$\begin{array}{ccccc} k_{\mathrm{eff}}^{\mathrm{MT}} & = & k_{m}\left(1-\frac{3f_{p}}{2+f_{p}}\right) & = & k_{m}\frac{2f_{m}}{3-f_{m}}\\ k_{\mathrm{eff}}^{\mathrm{DS}} & = & k_{m}{\left(1-f_{p}\right)}^{3/2} & = & k_{m}f_{m}^{3/2}\\ k_{\mathrm{eff}}^{\mathrm{SCS}} & = & k_{m}\left(1-\frac{3}{2}f_{p}\right) & = & k_{m}\frac{3f_{m}-1}{2} \end{array}$$

Note that in this case, a closed form solution for the differential scheme is obtained. The relation for the self consistent scheme is valid for $f_{m}$ greater than the so-called percolation threshold of $f_{m}=1/3$.

## Appendix B. Shape Factor for Spheroidal Pores

According to [20,36], the shape factor η depends on the aspect ratio γ, with γ=1 for a sphere, γ<1 and γ>1 corresponding to oblate and prolate shape, respectively:

(A11) $$\begin{array}{ccc} G & = & \frac{1}{\gamma \sqrt{1-\gamma^{2}}}\arctan\frac{\sqrt{1-\gamma^{2}}}{\gamma }\;\;\;\mathrm{for}\;\;\gamma <1, \end{array}$$

(A12) $$\begin{array}{ccc} G & = & \frac{1}{2\gamma \sqrt{\gamma^{2}-1}}\ln\frac{\gamma +\sqrt{\gamma^{2}-1}}{\gamma -\sqrt{\gamma^{2}-1}}\;\;\;\mathrm{for}\;\;\gamma >1, \end{array}$$

further

(A13) $$F=\frac{\gamma^{2}\left(1-G\right)}{2(\gamma^{2}-1)},$$

and, finally,

(A14) $$\eta =A_{1}+A_{2}/3,$$

with

(A15) $$A_{1}=\frac{1}{1-F}\;\mathrm{and}\;A_{2}=\frac{1-3F}{2F(1-F)}.$$

## Appendix C. Stress Concentration Factors in the Vicinity of Spherical Pores

Aligning the poles of a spherical pore with the direction of the far field uniaxial tensile stress σ, the polar angle 0≤θ≤π (angle with respect to polar axis) and the azimuthal angle −π≤ϕ≤π define the position on the pore surface. With radial coordinate *r* and pore radius *R*, according to [44], the principal stress state at r=R is given in radial direction as $\sigma_{rr}=0$, in the meridian direction as:

(A16) $$\frac{\sigma_{\theta \theta }}{\sigma }=\frac{27-15\nu }{2(7-5\nu )}-\frac{15}{\left(7-5\nu \right)}\cos^{2}\theta ;$$

and in the latitude direction as:

(A17) $$\frac{\sigma_{\phi \phi }}{\sigma }=-\frac{3(1-5\nu )}{2(7-5\nu )}-\frac{15\nu }{\left(7-5\nu \right)}\cos^{2}\theta ;$$

both a function of Poisson’s ratio ν and polar angle θ only, i.e., independent on pore radius *R*. The poles are characterized by biaxial compression with $\sigma_{\theta \theta }=\sigma_{\phi \phi }$ amounting to −3/14σ for ν=0, −1/2σ for ν = 0.2, and −7/6σ for ν = 0.5.

As regards the maximum tensile stress, the equator θ=π/2 is significant; for ν=0.2, the stress state is uniaxial with:

(A18) $$\sigma_{\theta \theta }=2\sigma \;\;\;and\;\;\;\sigma_{\phi \phi }=0;$$

for ν=0.5, biaxial tension with:

(A19) $$\sigma_{\theta \theta }=\frac{13}{6}\sigma =2.1\dot{6}\;\sigma \;\;\;and\;\;\;\sigma_{\phi \phi }=\frac{1}{2}\sigma ;$$

and for ν=0, tension/compression with:

(A20) $$\sigma_{\theta \theta }=\frac{27}{14}\sigma =1.928\ldots \sigma \;\;\;\mathrm{and}\;\;\;\sigma_{\phi \phi }=-\frac{3}{14}\sigma =-0.214\ldots \sigma .$$

## References

[1] Leonelli C., Kamseu E., Boccaccini D.N., Melo U.C., Rizzuti A., Billong N., Miselli P. (2007). Volcanic ash as alternative raw materials for traditional vitrified ceramic products. Adv. Appl. Ceram..

[2] Kamseu E., Boccaccini D.N., Sola A., Rizzuti A., Leonelli C., Melo C.U., Billong N. (2008). Sintering behaviour, microstructure and mechanical properties of low quartz content vitrified ceramics using volcanic ash. Adv. Appl. Ceram..

[3] Jin S., Choi J.W., Jeong C.M., Huh J.B., Lee S.H., Lee H., Yun M.J. (2019). Evaluating the wear of resin teeth by different opposing restorative materials. Materials.

[4] Vu V.A., Cloutier A., Bissonnette B., Blanchet P., Dagenais C. (2020). Steatite powder additives in wood-cement drywall particleboards. Materials.

[5] Kannaiyan S., Huang S.J., Rathnaraj D., Srinivasan S.A. (2022). Effect of ball-milled steatite powder on the latent heat energy storage properties and heat charging—Discharging periods of paraffin wax as phase change material. Micromachines.

[6] Ashurov M.K., Nuritdinov I., Saidakhmedov K.K., Ismailov S.K. (2021). Nature of the luminescence of SNC steatite ceramic. At. Energy.

[7] Lorenzoni F.C., Bonfante E.A., Valverde G.B., Coelho P.G., Bonfante G., Thompson V., Silva N.R. (2020). Effect of indenter material on reliability of all-ceramic crowns. J. Mech. Behav. Biomed. Mater..

[8] Nawafleh N., Bibars A.R., Al Twal E., Öchsner A. (2020). Influence of antagonist material on fatigue and fracture resistance of zirconia crowns. Eur. J. Dent..

[9] Perfler L., Peyker L., Hörtnagel M., Weinberger N., Pichler C., Traxl R., Lackner R. (2022). Pore space of steatite ceramics triggered by the allowance of natural fibers: High-resolution X-ray microscopy analysis and related thermo-mechanical properties. Mater. Des..

[10] Pichler C., Lackner R. (2012). Sesqui-power scaling of elasticity of closed-cell foams. Mater. Lett..

[11] Pichler C., Lackner R. (2013). Sesqui-power scaling of plateau strength of closed-cell foams. Mater. Sci. Eng. A.

[12] Pichler C., Traxl R., Lackner R. (2015). Power-law scaling of thermal conductivity of highly porous ceramics. J. Eur. Ceram. Soc..

[13] Traxl R., Pichler C., Lackner R. (2016). Thin-Shell Model for Effective Thermal and Electrical Conductivity of Highly Porous Closed-Cell Metal Foams. Transp. Porous Media.

[14] Parker W.J., Jenkins R.J., Butler C.P., Abbott G.L. (1961). Flash Method of Determining Thermal Diffusivity, Heat Capacity, and Thermal Conductivity. J. Appl. Phys..

[15] Cowan R.D. (1963). Pulse Method of Measuring Thermal Diffusivity at High Temperatures. J. Appl. Phys..

[16] Cape J.A., Lehman G.W. (1963). Temperature and Finite Pulse-Time Effects in the Flash Method for Measuring Thermal Diffusivity. J. Appl. Phys..

[17] Love A.E.H. (1959). A Treatise of the Mathematical Theory of Elasticity.

[18] Kohlhauser C., Hellmich C. (2013). Ultrasonic contact pulse transmission for elastic wave velocity and stiffness determination: Influence of specimen geometry and porosity. Eng. Struct..

[19] Kolsky H. (1953). Stress Waves in Solids.

[20] Sevostianov I., Kachanov M., Kachanov M., Sevostianov I. (2013). Non-interaction approximation in the problem of effective properties. Effective Properties of Heterogeneous Materials.

[21] Maxwell J.C. (1904). Treatise on Electricity and Magnetism.

[22] Kanaun S.K., Levin V.M., Markov K.Z. (1994). Effective field method in mechanics of matrix composite materials. Recent Advances in Mathematical Modelling of Composite Materials.

[23] Kanaun S.K. (2003). Dielectric properties of matrix composite materials with high volume concentrations of inclusions (effective field approach). Int. J. Eng. Sci..

[24] Markov K.Z. (2001). Justification of an effective field method in elasto-statics of heterogenous solids. J. Mech. Phys. Solids.

[25] Kanaun S., Levin V., Kachanov M., Sevostianov I. (2013). Effective field method in the theory of heterogeneous media. Effective Properties of Heterogeneous Materials.

[26] Hashin Z., Shtrikman S. (1962). A variational approach to the theory of the effective magnetic permeability of multiphase materials. J. Appl. Phys..

[27] Tanaka K., Mori T. (1972). Note on volume integrals of the elastic field around an ellipsoidal inclusion. J. Elast..

[28] Mori T., Tanaka K. (1973). Average stress in matrix and average elastic energy of materials with misfitting inclusions. Acta Metall..

[29] Zaoui A., Suquet P. (1997). Structural morphology and constitutive behaviour of microheterogeneous materials. Continuum Micromechanics, CISM Courses and Lectures No. 377.

[30] Zaoui A. (2002). Continuum micromechanics: Survey. J. Eng. Mech. (ASCE).

[31] Bruggeman D.A.G. (1935). Berechnung verschiedener physikalischer Konstanten von heterogenen Stoffen. I. Dielektrizitätskonstanten und Leitfähigkeiten [Determination of various physical constants of heterogenous media. I. Dielectric constants and conductivities]. Ann. Der Phys. Leipz..

[32] McLaughlin R. (1977). A study of the differential scheme for composite materials. Int. J. Eng. Sci..

[33] Norris A.N. (1985). A differential scheme for the effective moduli of composites. Mech. Mater..

[34] Eshelby J.D. (1957). The determination of the elastic field of an ellipsoidal inclusion, and related problems. Proc. R. Soc. Lond. A.

[35] Eshelby J.D., Sneddon I.N., Hill R. (1962). Elastic inclusions and inhomogeneities. Progress in Solid Mechanics 2.

[36] Sevostianov I., Kovacik J., Simancik F. (2006). Elastic and electric properties of closed-cell aluminum foams: Cross-property connection. Mater. Sci. Eng. A.

[37] Hershey A.V. (1954). The elasticity of an isotropic aggregate of anisotropic cubic crystals. J. Appl. Mech. (ASME).

[38] Kroener E. (1958). Berechnung der elastischen Konstanten des Vielkristalls aus den Konstanten des Einkristalls [Computation of the elastic constants of a polycrystal based on the constants of the single crystal]. Z. F. Phys..

[39] Dormieux L., Kondo D., Ulm F.J. (2006). Microporomechanics.

[40] Traxl R., Lackner R. (2015). Multi-level homogenization of strength properties of hierarchical-organized matrix-inclusion materials. Mech. Mater..

[41] Deng Z.Y., Fukasawa T., Ando M., Zhang G.J., Ohji T. (2001). Microstructure and mechanical properties of porous alumina ceramics fabricated by the decomposition of aluminum hydroxide. J. Am. Ceram. Soc..

[42] Jelitto H., Schneider G. (2018). A geometric model for the fracture toughness of porous materials. Acta Mater..

[43] Jelitto H., Schneider G.A. (2019). Fracture toughness of porous materials—Experimental methods and data. Data Brief.

[44] Goodier J.N. (1933). Concentration of stress around spherical and cylindrical inclusions and flaws. J. Appl. Mech. (ASME).

[45] Boccaccini A.R., Ondracek G., Mombello E. (1995). Determination of stress concentration factors in porous materials. J. Mater. Sci. Lett..

[46] Ondracek G. (1987). The quantitative microstructure field property correlation of multiphase and porous materials. Rev. Powder Metall. Phys. Ceram..

[47] Buchner T., Kiefer T., Königsberger M., Jäger A., Füssl J. (2021). Continuum micromechanics model for fired clay bricks: Upscaling of experimentally identified microstructural features to macroscopic elastic stiffness and thermal conductivity. Mater. Des..

[48] Buchner T., Königsberger M., Jäger A., Füssl J. (2022). A validated multiscale model linking microstructural features of fired clay brick to its macroscopic multiaxial strength. Mech. Mater..

[49] Timothy J.J., Meschke G. (2011). Micromechanics model for tortuosity and homogenized diffusion properties of porous materials with distributed micro-cracks. PAMM.

[50] Timothy J.J., Meschke G. (2015). Cascade continuum micromechanics model for the effective diffusivity of porous materials: Exponential hierarchy across cascade levels. PAMM.

[51] Timothy J.J., Meschke G. (2016). A cascade continuum micromechanics model for the effective elastic properties of porous materials. Int. J. Solids Struct..

[52] Timothy J.J., Meschke G. (2018). Effective diffusivity of porous materials with microcracks: Self-similar mean-field homogenization and pixel finite element simulations. Transp. Porous Media.

